# Draft Genome Sequence of Clostridium estertheticum subsp. *laramiense* DSM 14864^T^, Isolated from Spoiled Uncooked Beef

**DOI:** 10.1128/MRA.01275-19

**Published:** 2019-11-21

**Authors:** Nikola Palevich, Faith P. Palevich, Paul H. Maclean, Ruy Jauregui, Eric Altermann, John Mills, Gale Brightwell

**Affiliations:** a AgResearch Limited, Grasslands Research Centre, Palmerston North, New Zealand; b Riddet Institute, Massey University, Palmerston North, New Zealand; Indiana University, Bloomington

## Abstract

Clostridium estertheticum subsp. *laramiense* type strain DSM 14864 (ATCC 51254) was isolated from vacuum-packaged refrigerated spoiled beef. This report describes the generation and annotation of the 5.0-Mb draft genome sequence of *C. estertheticum* subsp. *laramiense* DSM 14864^T^.

## ANNOUNCEMENT

Clostridium estertheticum subsp. *laramiense* DSM 14864^T^ (ATCC 51254^T^) is a Gram-positive, spore-forming, slow-growing psychotrophic anaerobe that was originally isolated from vacuum-packaged refrigerated spoiled beef at the University of Wyoming (Laramie, WY) (1). Numerous bacterial species belonging to the genus *Clostridium* have been recognized as causative agents of blown pack spoilage (BPS) in vacuum-packed meat products, and DSM 14864^T^ was selected for genome sequencing to examine its role in BPS. Phylogenetic analysis based on 16S rRNA gene sequence data placed DSM 14864^T^ within the Clostridium estertheticum species, being 99% similar to the *C. estertheticum* type strain DSM 8809 (ATCC 51377) (2). On the basis of genetic and phenotypic properties, *C. laramiense* and *C. estertheticum* were united and reclassified as *C. estertheticum* subsp. *laramiense* subsp. nov., represented by strain DSM 14864^T^ (ATCC 51254^T^) (3).

Strain DSM 14864^T^ was acquired from the Leibniz Institute DSMZ-German Collection of Microorganisms and Cell Cultures and cultured anaerobically at 10°C in prereduced peptone-yeast extract-glucose-starch (PYGS) broth (4). Genomic DNA was extracted using a modified phenol-chloroform procedure (5) and mechanically sheared using a Nebulizer instrument (Invitrogen) to select fragments of approximately 550 bp. A DNA library was prepared using the Illumina TruSeq Nano method and sequenced on the Illumina MiSeq platform with the 2 × 250-bp paired-end (PE) reagent kit v2, producing a total of 4,394,200 PE raw reads. The quality of the raw reads was checked in FastQC v0.11.5 (https://www.bioinformatics.babraham.ac.uk/projects/fastqc/), and the reads were trimmed with Trimmomatic v0.39 (http://www.usadellab.org/cms/?page=trimmomatic) and assembled using the A5-miseq pipeline v20169825, with standard parameters (6). The *de novo* assembly of DSM 14864^T^ produced 85 scaffolds with 202× coverage and an *N*_50_ value of 226,678 bp, with the largest scaffold length being 633,186 bp in size. The draft genome sequence is composed of 5,000,223 bp, with a G+C content of 30.5%. A total of 4,743 putative protein-coding genes (PCGs) were predicted, along with 89 tRNA, 26 rRNA, and 243 noncoding RNA (ncRNA) elements using GAMOLA2 (7). In addition, DIAMOND v0.9.21.122 (8) and InterProScan v5.36-75.0 (9) were used to search the NCBI nr database. The resulting protein set was imported into Blast2GO, as implemented in the OmicsBox software package v1.1.164 (10), with which gene ontology terms and final annotations were assigned to each protein. All bioinformatics analyses were performed using default settings and parameters.

Carbohydrate-Active enZyme (CAZy) (11) profiling was analyzed using dbCAN2 (12) and revealed that the DSM 14864^T^ genome is predicted to encode 61 glycoside hydrolase (GH), 34 glycosyltransferase (GT), 6 polysaccharide lyase (PL), 11 carbohydrate esterase (CE), and 20 carbohydrate-binding protein module (CBM) families. Overall, approximately 3% of the DSM 14864^T^ genome (132 coding sequences [CDSs]) is predicted to encode either secreted or intracellular proteins dedicated to carbohydrate and polysaccharide degradation. Interestingly, the enzymatic profiles of DSM 14864^T^ and the well-characterized strain *C. estertheticum* DSM 8809^T^ (ATCC 51377^T^) (2) are almost identical, as both are equipped to utilize many oligosaccharides and monosaccharides as substrates for growth and encode a large repertoire of enzymes predicted to metabolize complex insoluble polysaccharides such as xylan and pectin.

The genome sequence of Clostridium estertheticum subsp. *laramiense* strain DSM 14864^T^ (ATCC 51254^T^) reported here is a valuable resource for future studies investigating the bacterial genetic mechanisms associated with BPS. In order to improve the phylogenetic resolution of the genus *Clostridium* and improve our limited knowledge of meat spoilage caused by C. estertheticum, future efforts should focus on the generation of complete genomes across a wider range of Clostridium species.

### Data availability.

The genome sequence and associated data for Clostridium estertheticum subsp. *laramiense* type strain DSM 14864 (ATCC 51254) were deposited under GenBank accession number WBOE00000000, BioProject number PRJNA574489, and Sequence Read Archive (SRA) accession number SRR10193440.

## References

[1] Kalchayanand N, Ray B, Field RA, Johnson MC 1989 Spoilage of vacuum-packaged refrigerated beef by *Clostridium*. J Food Prot 52:424–426. doi:10.4315/0362-028X-52.6.424. 31003305

[2] Yu Z, Gunn L, Brennan E, Reid R, Wall PG, Gaora PÓ, Hurley D, Bolton D, Fanning S 2016 Complete genome sequence of *Clostridium estertheticum* DSM 8809, a microbe identified in spoiled vacuum packed beef. Front Microbiol 7:1764. doi:10.3389/fmicb.2016.01764. 27891116 PMC5104964

[3] Spring S, Merkhoffer B, Weiss N, Kroppenstedt RM, Hippe H, Stackebrandt E 2003 Characterization of novel psychrophilic clostridia from an Antarctic microbial mat: description of *Clostridium frigoris* sp. nov., *Clostridium lacusfryxellense* sp. nov., *Clostridium bowmanii* sp. nov. and *Clostridium psychrophilum* sp. nov. and reclassification of *Clostridium laramiense* as *Clostridium estertheticum* subsp. *laramiense* subsp. nov. Int J Syst Evol Microbiol 53:1019–1029. doi:10.1099/ijs.0.02554-0. 12892121

[4] Lund BM, Graham AF, George SM, Brown D 1990 The combined effect of incubation temperature, pH and sorbic acid on the probability of growth of non-proteolytic, type B *Clostridium botulinum*. J Appl Bacteriol 69:481–492. doi:10.1111/j.1365-2672.1990.tb01539.x. 2292514

[5] Bouillaut L, McBride SM, Sorg JA 2011 Genetic manipulation of *Clostridium difficile*. Curr Protoc Microbiol Chapter 9:Unit 9A.2. 10.1002/9780471729259.mc09a02s20 PMC3615975 21400677

[6] Coil D, Jospin G, Darling AE 2015 A5-miseq: an updated pipeline to assemble microbial genomes from Illumina MiSeq data. Bioinformatics 31:587–589. doi:10.1093/bioinformatics/btu661. 25338718

[7] Altermann E, Lu J, McCulloch A 2017 GAMOLA2, a comprehensive software package for the annotation and curation of draft and complete microbial genomes. Front Microbiol 8:346. doi:10.3389/fmicb.2017.00346. 28386247 PMC5362640

[8] Buchfink B, Xie C, Huson DH 2015 Fast and sensitive protein alignment using DIAMOND. Nat Methods 12:59. doi:10.1038/nmeth.3176. 25402007

[9] Jones P, Binns D, Chang H-Y, Fraser M, Li W, McAnulla C, McWilliam H, Maslen J, Mitchell A, Nuka G, Pesseat S, Quinn AF, Sangrador-Vegas A, Scheremetjew M, Yong S-Y, Lopez R, Hunter S 2014 InterProScan 5: genome-scale protein function classification. Bioinformatics 30:1236–1240. doi:10.1093/bioinformatics/btu031. 24451626 PMC3998142

[10] Conesa A, Götz S, García-Gómez JM, Terol J, Talón M, Robles M 2005 Blast2GO: a universal tool for annotation, visualization and analysis in functional genomics research. Bioinformatics 21:3674–3676. doi:10.1093/bioinformatics/bti610. 16081474

[11] Cantarel BL, Coutinho PM, Rancurel C, Bernard T, Lombard V, Henrissat B 2009 The Carbohydrate-Active EnZymes database (CAZy): an expert resource for glycogenomics. Nucleic Acids Res 37:D233–D238. doi:10.1093/nar/gkn663. 18838391 PMC2686590

[12] Zhang H, Yohe T, Huang L, Entwistle S, Wu P, Yang Z, Busk PK, Xu Y, Yin Y 2018 dbCAN2: a meta server for automated carbohydrate-active enzyme annotation. Nucleic Acids Res 46:W95–W101. doi:10.1093/nar/gky418. 29771380 PMC6031026

